# Polyphosphate-kinase-1 dependent polyphosphate hyperaccumulation for acclimation to nutrient loss in the cyanobacterium, *Synechocystis* sp. PCC 6803

**DOI:** 10.3389/fpls.2024.1441626

**Published:** 2024-07-31

**Authors:** Norihiro Sato, Mizuki Endo, Hiroki Nishi, Shoko Fujiwara, Mikio Tsuzuki

**Affiliations:** School of Life Sciences, Tokyo University of Pharmacy and Life Sciences, Hachioji, Tokyo, Japan

**Keywords:** cyanobacteria, polyphosphate, polyphosphate kinase1, PPK1, sulfur starvation, *Synechocystis*

## Abstract

Polyphosphate is prevalent in living organisms. To obtain insights into polyphosphate synthesis and its physiological significance in cyanobacteria, we characterize *sll0290*, a homolog of the polyphosphate-kinase-1 gene, in the freshwater cyanobacterium *Synechocystis* sp. PCC 6803. The Sll0290 protein structure reveals characteristics of Ppk1. A *Synechocystis sll0290* disruptant and *sll0290*-overexpressing *Escherichia coli* transformant demonstrated loss and gain of polyphosphate synthesis ability, respectively. Accordingly, *sll0290* is identified as *ppk1*. The disruptant (Δ*ppk1*) grows normally with aeration of ordinary air (0.04% CO_2_), consistent with its photosynthesis comparable to the wild type level, which contrasts with a previously reported high-CO_2_ (5%) requirement for Δ*ppk1* in an alkaline hot spring cyanobacterium, *Synechococcus* OS-B’. *Synechocystis* Δ*ppk1* is defective in polyphosphate hyperaccumulation and survival competence at the stationary phase, and also under sulfur-starvation conditions, implying that sulfur limitation is one of the triggers to induce polyphosphate hyperaccumulation in stationary cells. Furthermore, Δ*ppk1* is defective in the enhancement of total phosphorus contents under sulfur-starvation conditions, a phenomenon that is only partially explained by polyphosphate hyperaccumulation. This study therefore demonstrates that in *Synechocystis*, *ppk1* is not essential for low-CO_2_ acclimation but plays a crucial role in dynamic P-metabolic regulation, including polyP hyperaccumulation, to maintain physiological fitness under sulfur-starvation conditions.

## Introduction

Polyphosphate (polyP) is a linear polymer consisting of three to hundreds of inorganic phosphate residues linked by high-energy phosphoanhydride bonds, and is prevalent in prokaryotes and eukaryotes, serving as reservoirs of phosphorus (P) and energy (Rao et al., 2009). PolyP is stored as granules called polyP bodies, which prokaryotic cells house in the cytoplasm in most cases or acidocalcisomes, acidic organelles rich in calcium, in a few cases, and are usually present in acidocalcisomes in eukaryotic cells (Docampo et al., 2005; Rao et al., 2009; Denoncourt and Downey, 2021). Microorganisms have long been known to hyperaccumulate polyP under stress conditions such as entry into the stationary phase, which accompanies multiple stresses including nutrient depletion (Terry and Hooper, 1970; Kim et al., 1998; Komine et al., 2000; Chen et al., 2002; Zhang et al., 2005; Rao et al., 2009), and specific-nutrient limited conditions, such as starvation for amino acids in non-photosynthetic prokaryotes (Harold, 1966; Ault-Riché et al., 1998; Racki et al., 2017), and nitrogen (N) or sulfur (S) in photosynthetic microbes including cyanobacteria and algae (Harold, 1966; Grillo and Gibson, 1979; Lawry and Jensen, 1979; Kuesel et al., 1989; Aksoy et al., 2014; Ota et al., 2016; Goodenough et al., 2019; Sanz-Luque et al., 2020a). Other typical cases include repletion with Pi after its limitation, which induces polyP hyperaccumulation designated as polyP overplus phenomenon (Sanz-Luque et al., 2020a). Conversely, polyP is degraded in microbes when relived from above stresses that induce polyP hyperaccumulation or exposed simply to P starvation (Goodenough et al., 2019; Li and Dittrich, 2019; Hiyoshi et al., 2021).

PolyP is synthesized by polyP kinase (PPK), which elongates the phosphate chain through successive addition of γ-phosphate groups of ATP, whereas it is degraded by exopolyphosphatase (PPX), which releases the terminal phosphate through hydrolysis (Harold, 1966; Rao et al., 2009). The gene for PPK (*ppk1*) was initially cloned in *E. coli*, followed by the discovery of its homologs in many prokaryotes (Akiyama et al., 1992; Rao et al., 2009). In some prokaryotic groups, including *Pseudomonas aeruginosa*, the *ppk2* gene was later found, encoding PPK that is structurally unrelated to Ppk1 and utilizes GTP as well as ATP as the substrate (Zhang et al., 2002; Rao et al., 2009). Ppk1 and Ppk2 can also catalyze reverse reactions for ATP synthesis, and ATP or GTP synthesis, respectively. Ppk1 favors polyP synthesis over ATP synthesis, while Ppk2 shows a preference for ATP/GTP synthesis over polyP synthesis (Rao et al., 2009; Bowlin and Gray, 2021). The *ppx* gene, first cloned in *E. coli*, is prevalent in prokaryotes (Akiyama et al., 1993). The *ppk1*, *ppk2*, and *ppx* genes are generally absent in eukaryotes; however, some limited groups of lower eukaryotes exceptionally possess *ppk1* homologs (Whitehead et al., 2013). Particularly, in a slime mold, *Dictyostelium discoideum*, its eukaryotic homolog was functionally identified as *ppk1* (Zhang et al., 2005).

Following the identification of *ppk* and *ppx*, mutants of these genes have been generated and characterized in a variety of non-photosynthetic microorganisms, deepening the understanding of the commitment of these genes in polyP metabolism *in vivo* and the physiological roles of polyP metabolism. Regarding polyP synthesis, *ppk1* is a main determinant of the cellular polyP content at the steady-state and also at nutrient-stress induced hyperaccumulation level (Chen et al., 2002; Zhang et al., 2005). Above all, *ppk1* is essential for cellular acclimation not only to the polyP-hyperaccumulation stress but also other stresses such as heat, oxidation, and high osmosis (Rao and Kornberg, 1996; Kim et al., 1998; Kuroda et al., 1999; Rao et al., 2009). Notably, *ppk1* is necessary for motility and biofilm formation, underlying virulence in pathogenic species like *P. aeruginosa* (Rashid and Kornberg, 2000; Rashid et al., 2000a, 2000b). Meanwhile, *ppx* as well as *ppk1* was crucial for motility and biofilm formation, demonstrating that dynamic regulation of polyP metabolism is crucial for these physiological processes (Gallarato et al., 2014; Denoncourt and Downey, 2021; Tiwari et al., 2022).

Many cyanobacteria possess both *ppk1* and *ppk2* homologs and *ppx* one; however, information is limited on their enzymatic and physiological roles. It was first reported in cyanobacteria that a *ppk1* homolog could not be completely disrupted in *Synechocystis* sp. PCC 6803 (hereafter referred to as *Synechocystis*), implying some essential role of polyP synthesis (Gómez-García et al., 2003). Later, in *Synechococcus* OS-B’ (hereafter referred to as *Synechococcus*), a complete disruptant as to its *ppk1* homolog was generated to show a defect in polyP hyperaccumulation, thereby, the homolog being functionally identified as *ppk1* (Gómez-García et al., 2013). Simultaneously, pleiotropic effects were exerted in the *Synechococcus* disruptant, Δ*ppk1*, including the inability of cells to grow with the aeration of ordinary air containing 0.04% CO_2_ (low-CO_2_ conditions) (Gómez-García et al., 2013). Since the *Synechococcus* Δ*ppk1*, similar to the WT, grew vigorously with the aeration of CO_2_-enriched air (5% CO_2_, high-CO_2_ conditions), it might be interpreted that *ppk1* was dispensable for cell growth under nutrient-rich conditions (CO_2_-rich in this case), as is often the case with non-photosynthetic microbes (Crooke et al., 1994; McMeechan et al., 2007; Rashid et al, 2000b; Zhang et al., 2005). Concerning *ppx*, its *Synechocystis* disruptant (Δ*ppx*) showed normal cell growth under low CO_2_ conditions but retarded one, specifically under P-starvation (-P) conditions (Hiyoshi et al., 2021). The *ppx* gene is thus essential in *Synechocystis* for cellular acclimation to -P stress but not to low-CO_2_ one. Meanwhile, the expression level of *ppk1* peaked in the evening over a diel cycle in *Synechococcus* whereas the *ppx* homolog was expressed most strongly in the early morning, which implied the cruciality of the regulatory polyP metabolism for optimized cell growth in the diel cycle (Gómez-García et al., 2013).

To gain deeper insights into polyP metabolism and its physiological significance in cyanobacteria, this study generated a complete disruptant of the *ppk1* homolog, *sll0290*, in *Synechocystis*. The characterization of the disruptant led us to identify *sll0290* as *ppk1* and to find that *ppk1* is not required for cell growth under low CO_2_ conditions but is essential for proper maintenance of physiological fitness upon entry into the stationary phase that would accompany nutrient depletion stresses, or during the imposition of -S stress.

## Materials and methods

### Cyanobacterial strains and growth conditions

The cyanobacterial strains used were *Synechocystis* and its mutant, in which the genomic copies of *ppk1* were completely disrupted (Δ*ppk1*, see below). The cells were cultured at 30°C in glass tubes containing BG11 medium, with illumination (60 μmol photons·m^-2^·s^-1^) and ordinary-air aeration, as previously described (Hiyoshi et al., 2021). For polyP hyperaccumulation, *Synechocystis* WT or Δ*ppk1* culture was initially adjusted to OD_730_ = 0.2 in a modified BG11 medium with sulfate-free (-S) and phosphate (Pi)-surplus (++P: 1 mM Pi, c.f., 0.22 mM Pi normally in BG11 medium). The culture was then grown for 2 days, following the procedure described previously (Lawrence et al., 1998). Otherwise, -S was simply imposed on the cells by culturing in -S BG11 medium, as previously described (Hirai et al., 2019). OD_730_ in the culture, and chlorophyll (Chl) and phycobilisome (PBS) contents were determined using a spectrophotometer (DU 640, Beckman) for investigation of cell growth, as described previously (Hiyoshi et al., 2021).

### Extraction of polyP bodies, and measurement of their constituent Pi contents

PolyP bodies were isolated from *Synechocystis* cells cultured under -S/++P or +S/++P conditions, as described above. PolyP bodies were isolated from cells, according to the method described by Shi et al. (2003). The culture (40 mL) was subjected to centrifugation (3,500 g for 15 min at 4°C) to recover cells as a pellet, which was then digested by treating with 1 mL alkaline hypochlorite reagent (5.4%) for 45 min at 25°C. The digested-cell suspension was centrifugated to collect residues, which were washed twice with alkaline hypochlorite reagent. PolyP bodies were extracted from the residues twice with distilled water (0.5 mL) and precipitated by centrifugation (14,000 g for 10 min at 4°C) after the addition of ethanol (9 mL). Isolated polyP bodies were degraded into Pi by hydrolysis, and their constituent Pi contents were spectroscopically measured (Hiyoshi et al., 2021).

### ^31^P-NMR measurement

Cells cultured under -S/++P or +S/++P conditions were harvested by centrifugation, and then were resuspended in 0.5 M EDTA with adjustment of Chl concentration to 5 mg·mL^-1^. ^31^P-NMR spectra of the cell suspension were measured with a Bruker PDX400 NMR spectrometer, and a 5 mm diameter QNP probe head operating at 162.2 MHz in the pulsed Fourier transform mode, as described previously (Kobayashi et al., 2005). A total of 3,072 scans were accumulated for each spectrum with line broadening of 5.0 Hz. Chemical shifts were measured in ppm relative to external 85% Pi (w/w) (Kobayashi et al., 2005). Signal peaks were assigned to soluble polyP and Pi/phosphate monoesters, according to the report by Yang et al. (1993).

### Disruption of sll0290 in Synechocystis

A DNA fragment covering the C-terminal half of *Sll0290*, a homolog of *Ppk1*, in *Synechocystis* was amplified by Ex-taq DNA polymerase (Takara, Japan) with primers 1 (5’- TGACCCTGTACCGCACTTCG-3’) and 2 (5’-TCAAACTGAACGTAGTTCCG-3’), as previously described (Hiyoshi et al., 2021). A product of 1.0 kbp was ligated to the pGEM T-EASY vactor (Promega) to generate the plasmid, which was then cut at two *Bal*I sites present in the center of the DNA region corresponding to the PCR product, followed by insertion of the spectinomycin-resistant gene cassette (*Spc^R^*). The resultant plasmid containing the disrupted *sll0290* was used to transform wild-type (WT) cells of *Synechocystis* by homologous recombination, as described previously (Hiyoshi et al., 2021). The disruption of *sll0290* was confirmed by PCR with two primer sets: one consists of primers 1 and 2 whereas the other constitutes primers 2 and 3 (5’-CGGACCATTGTTTAAATGGG -3’).

### Overexpression of the *ppk1* gene in *Escherichia coli*

For amplification of the coding region of the *ppk1* gene in *Synechocystis*, PCR was performed with primers 4 (5’-ATCCAAGCTATGCCCTCTGC-3’) and 5 (5’-ACTGAACGTAGTTCCGGGTC-3’), as described above, with the change of Ex-Taq DNA polymerase to KOD-plus one (Toyobo, Japan). A product of 2.2 kbp was ligated to the pET15b vector (Promega) that had been blunted after cut by *Nco*I site. The resultant plasmid was introduced into BL21 competent cells of *E. coli*, and thereafter the transformants were used for induction of the *Synechocystis ppk1* gene expression, through addition of 1 mM IPTG, according to the manufacture’s protocol (Promega).

### Microscopic observation of polyP bodies in *Synechoystis* or *E. coli* cells

For fluorescence microscopic observations, *Synechocystis* or *E. coli* cells were fixed in a culture through addition of glyceraldehyde (1%), thereafter frozen in liquid-nitrogen and thawed in hands. The cells were then stained with 4’, 6-diamidino-2-phenylindole (DAPI, 50 µg/ml) and observed under a fluorescence microscope (BX-FLA, Olympus Optical Co., Tokyo, Japan) with the use of a 340-390 nm excitation filter. Meanwhile, for electron microscopic analysis, *Synechocystis* cells were fixed in a culture through addition of glutaraldehyde (2.5%) and were harvested by centrifugation (3,500 g for 15 min at 4°C). The cells were washed in 50 mM phosphate buffer (pH 7.0), and were fixed for 1 h in the phosphate buffer containing glutaraldehyde (2.5%). The glutaraldehyde-fixed cells were submitted to Japan Electron Optics Laboratory (JEOL) for contact research, where the cells were subjected to transmission electron microscopy (TEM) with JEM-1010 after post-fixation in 2% OsO_4_.

### Statistics

The significance of differences was evaluated using a two-sided Student’s *t*-test.

## Results

### Structural characterization of Sll0290 as an ortholog to known Ppk1 proteins

**Figure 1:**
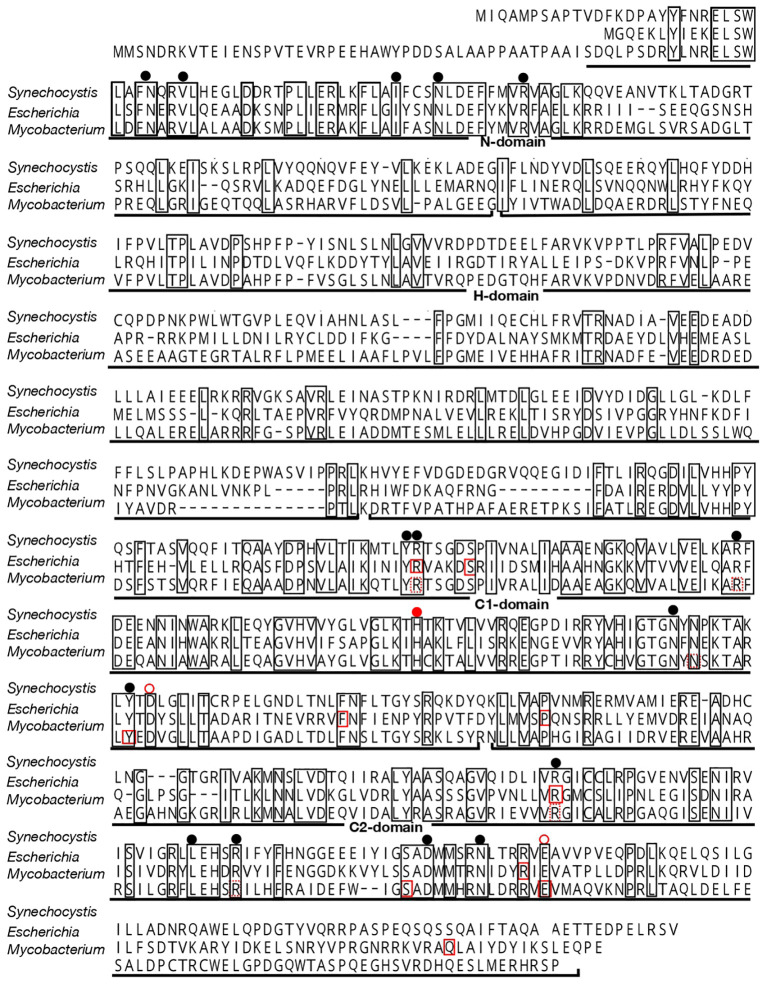
Alignment of the amino acid sequence of Sll0290 with those of Ppk1 proteins from *E. coli and M. tuberculosis*. Amino acid residues conserved among these three sequences are enclosed in squares. Solid-red rectangles indicate amino acid residues sequences that are responsible for both polyP and ATP syntheses, while dashed-red rectangles denote those for ATP synthesis in *E. coli and M. tuberculosis* (Tzeng and Kornberg, 2000; Mittal et al., 2011). Black circles indicate positions of amino acid residues involved in ATP binding in polyP synthesis in *E. coli* (Zhu et al., 2005). A closed red circle represents the His residue that is autophosphorylated for polyP synthesis in *E. coli* and *M. tuberculosis*, while open red circles indicate amino acid residues involved in the autophosphorylation in *E. coli* (Zhu et al., 2005; Mittal et al., 2011).

In general, *ppk1* acts as a key player in polyP synthesis, and its homologs are found mainly as a single copy in the genomes of respective cyanobacterial strains (0, 1, and 2-4 copies in 13, 227 and 37 strains, respectively; **Supplementary Table 1**), the nucleotide sequences of which have been thus far determined (CyanoCyc, https://cyanocyc.org/). In *Synechocystis*, *sll0290* is annotated as a sole *ppk1*, however, no structural characterization has been reported regarding its protein product. Firstly, we characterized the structure of Sll0290 by aligning its amino acid sequence with those of known Ppk1 proteins from other organisms. The amino acid sequence of Sll0290 showed 37% and 49% identity to Ppk1 proteins of *E. coli* and *Mycobacterium tuberculosis*, respectively (**Figure 1**). Accordingly, Sll0290 consisted of four regions, corresponding to characteristic domains revealed in the X-ray structure of *E. coli* Ppk1 (Zhu et al., 2005): the N-domain, a highly conserved region located at the N-terminus; the H-domain, with the lowest degree of homology, which interacts with the C1-domain for Ppk1 dimerization; and the C1- and C2-domains, two other highly conserved regions at the C-terminus. Furthermore, amino acid residues responsible for constructing the ATP-binding pocket (Zhu et al., 2005) and/or those found necessary for functioning of PPK and/or polyP: ADP phosphotransferase (Kumble et al., 1996; Tzeng and Kornberg, 2000; Mittal et al., 2011) were well conserved in Sll0290 (**Figure 1**). These structural features strongly suggested that *sll0290* encodes Ppk1.

### Functional identification of *sll0290* as encoding the polyP kinase

**Figure 2:**
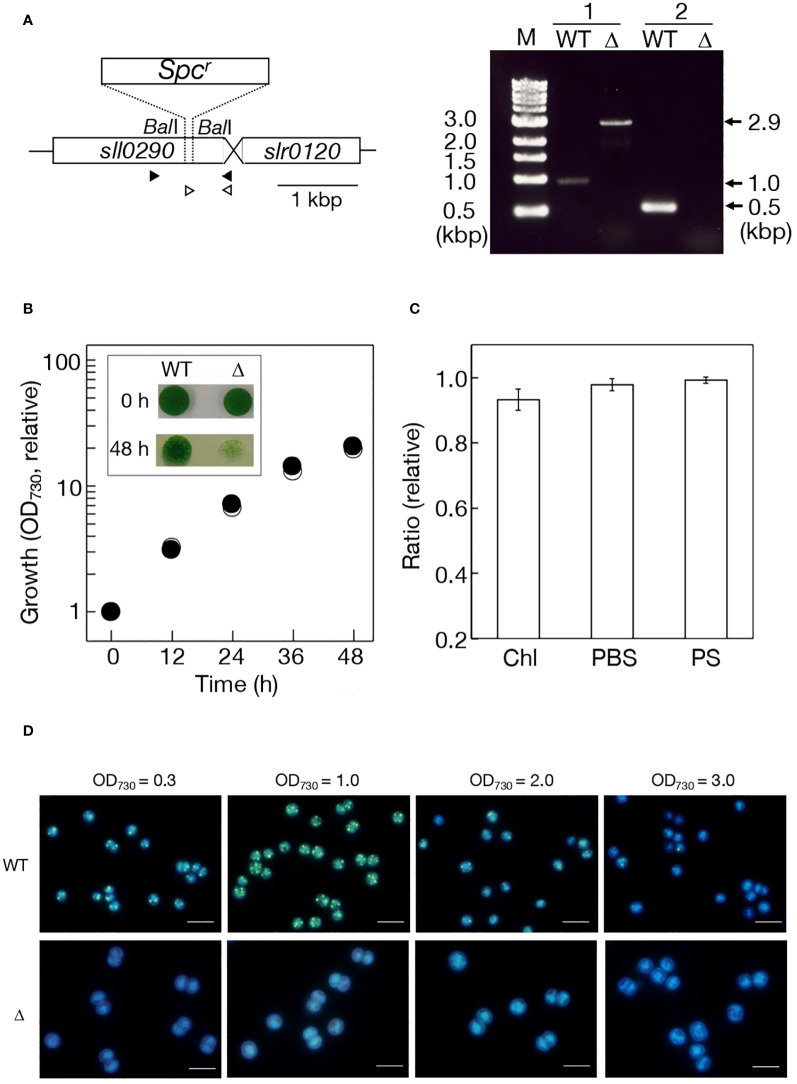
Effects of *sll0290* disruption on cell growth, photosynthesis, and polyP body accumulation. **(A)** Disruption strategy of *sll0290* through insertion of the spectinomycin resistant gene cassette (*Spc^r^*). Left, a DNA region of 0.2 kbp between two *Bal*I sites in *sll0290* was replaced by *Spc^r^*. Right, *sll0290* disruption was confirmed by genomic DNA PCR. Primer sets 1 (closed arrowheads) and 2 (open arrowheads) were used. Note that only a 2.9-kbp DNA fragment was amplified in Δ*sll0290* with the primer set 1, corresponding to the disrupted size of *sll0290*, and that no amplified DNA fragment appeared with the primer set 2 in Δ*sll0290*, compatible with the loss of the 0.2-kbp Ball DNA fragment. **(B)** Cell growth measured by increases in OD_730_ values in the culture. Shown are values relative to that in the WT culture at 0 h (0.10). Inset, cell viability on the BG11 agar plate. Liquid cultures at 0 h (precultures at the log phase) and 48 h were respectively diluted to OD_730_ = 0.1 in the BG11 medium, thereafter, the diluted cultures of 10 μL placed on an agar plate for subsequent cell growth. Open and closed circles indicate WT and Δ*sll0290*, respectively. **(C)** Effects of *sll0290* disruption on photosynthesis. Respective contents of Chl and PBS, and photosynthesis (PS) in Δ*sll0290* are shown relative to the WT (Chl, 7.08 μg·OD_730_^-1^·mL^-1^; PBS, 0.270 OD_730_^-1^·mL^-1^; PS, 322 μmol O_2_· mg Chl^-1^·h^-1^). **(D)** Fluorescence-microscopic images of WT and Δ*sll0290* cells stained with DAPI. Only early-stationary cells in the WT (OD_730_ = 1.0 corresponding to 10 in relative growth in **(B)**) exhibited strong green fluorescence emitted from polyP bodies in the background of blue fluorescence from DNA and RNA. Bars indicate 5 μm. The values indicated in **(B, C)** are the averages ± SD from three biological replicates, with SD bars in **(B)** hidden within symbols.

To investigate the catalytic function of *Sll0290*, we disrupted *sll0290* in the genome in *Synechocystis* by replacing a partial region of *sll0290* with the *Spc^R^* cassette. Genomic DNA PCR confirmed the disruption of *sll0290* in all genomic DNA copies (**Figure 2A**). The disruptant (Δ*sll0290*), similar to the WT, exhibited vigorous growth in liquid culture with ordinary-air aeration (**Figure 2B**), with no deleterious effects on the cellular content of Chl or phycobilisome, or photosynthesis (**Figure 2C**). These results demonstrated that *sll0290* is dispensable for the construction and functionality of the photosynthetic machinery, and inevitably for cell growth with ordinary-air aeration. Fluorescence-microscopic observations demonstrated DAPI-stained polyP bodies in logarithmically growing WT cells, contrastingly, no such structure observed in the Δ*sll0290* counterparts (**Figure 2D**). Notedly, WT cells exhibited a significant increase in polyP bodies upon entry into the stationary phase (OD_730_ = 1.0), followed by a subsequent decrease to levels lower than those initially observed as the stationary phase progressed (OD_730_ = 2.0 or 3.0). In contrast, Δ*sll0290* cells showed no discernible appearance of polyP bodies throughout the stationary phase (**Figure 2D**). These results indicated that *Synechocystis* cells increased polyP-body contents temporarily during the transition from logarithmic to stationary growth phases, and that *sll0290* is responsible for polyP-body accumulation at a low steady-state level, and also at an early-stationary-phase induced high level.

**Figure 3:**
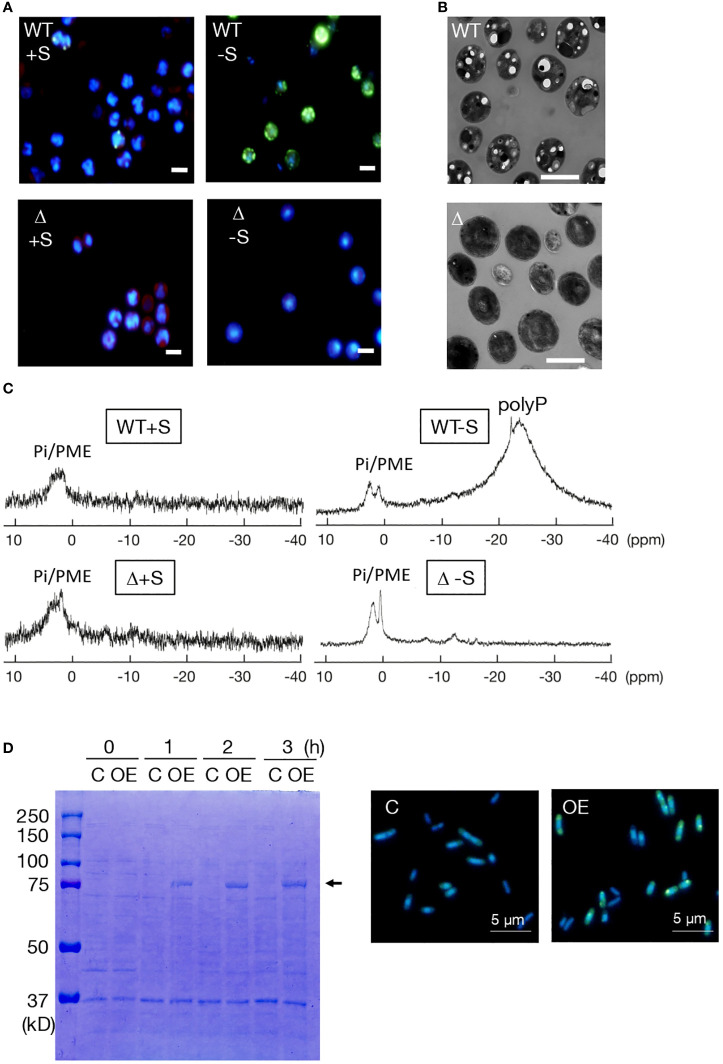
Dependency of polyP accumulation on *sll0290* in *Synechocystis* and *E coli*. **(A)** Fluorescence-microscopic images of WT and Δ*sll0290* cells stained with DAPI. Cells starved for S for 2 days in the presence of surplus Pi (1 mM Pi) hyperaccumulated polyP bodies in the WT but not in Δ*sll0290*; both WT and Δ*sll0290* showed the lack of polyP body hyperaccumulation when cultured with S repletion in 1 mM Pi. Bars indicate 2 μm. **(B)** Electron-microscopic images of WT and Δ*sll0290* cells starved for S for 2 days. Note that only WT cells exhibited several low-electron density holes, which would represent polyP bodies. Bars indicate 2 μm. **(C)** ^31^P-NMR spectra of the WT and Δ*sll0290* cells cultured with or without S, in the presence of 1 mM Pi. Signal peaks were assigned to soluble polyP and Pi/phosphate monoesters, according to the report by Yang et al. (1993). **(D)** Overexpression of *sll0290* in *E coli* cells. Left, SDS-PAGE of total proteins extracted from *E coli* cells transformed with the *sll0290* overexpression vector (OE) and those with the empty vector **(C)**. The arrow indicates an IPTG-induced protein. Right, fluorescence-microscopic images of OE and C cells stained with DAPI. Note that only OE cells exhibited hyperaccumulation of polyP bodies. Bars indicate 5 μm.

-S as well as entry into stationary phase is the well-known stressor for induction of polyP hyperaccumulation in photosynthetic microbes, including cyanobacteria. Here, the conditions of -S with surplus P (++P: 1 mM Pi, c.f., 0.22 mM normally) were used for investigation of polyP-body hyperaccumulation in *Synechocystis* (Lawrence et al., 1998). Fluorescence-microscopic images exhibited -S-induced polyP-body hyperaccumulation in the WT, and no polyP-body accumulation in Δ*sll0290* irrespective of +S/++P or -S/++P (**Figure 3A**). Consistently, transmission electron microscopic images displayed that -S/++P cells in WT contained several large spots of low electron density, which would represent traces of polyP bodies that were lost during ultra-thin sectioning (Lawry and Jensen, 1979; Hackenberg et al., 2012; Moura et al., 2019), whereas such structure was absent in Δ*sll0290* (**Figure 3B**). Therefore, it was found that *ppk1* is responsible for the -S-induced polyP-body hyperaccumulation by fluorescence- and electron-microscopy.

PolyP bodies were then isolated from WT and Δ*sll0290* cells, subsequently subjected to chemical Pi quantitation (**Table 1**). Under +S/++P conditions, polyP bodies amounted at 0.60 fmol P·cell^-1^ in WT, but at a significantly lower level of 0.04 fmol P·cell^-1^ in Δ*ppk1* (**Table 1**). Besides, -S caused WT cells to increase the polyP body content by 12.5-fold, consistent with the previous report (Lawrence et al., 1998); in contrast, Δ*sll0290* exhibited little stimulatory effect of -S on the polyP accumulation. These results, compatible with those obtained through above microscopic observations, strengthened our thought that *sll0290* is a key player to maintain the polyP-body level, irrespective of the extent of its accumulation level. Besides polyP bodies or insoluble polyP, soluble polyP was found to accumulate in the WT cells with -S/++P but not in those with +S/++P, through *in vivo* ^31^P NMR spectroscopic analysis (**Figure 3C**), compatible with the previous report (Lawrence et al., 1998). However, Δ*sll0290* cells showed no apparent signal of soluble polyP under either of these two conditions. Therefore, *sll0290* is responsible for -S-inducible accumulation of soluble polyP as well as that of polyP bodies.

**Table 1:** Effects of Δ*sll0290* on polyP accumulation.

+S/++P			-S/++P	
	WT	Δ*sll0290*	WT	Δ*sll0290*
fmol Pi·cell^-1^				
polyP	0.60 ± 0.28	0.04 ± 0.02*	7.50 ± 6.14	0.06 ± 0.04*
Pi	0.45 ± 0.22	0.75 ± 0.24	0.15 ± 0.11	0.56 ± 0.31
Total P	6.79 ± 2.12	3.12 ± 1.46*	36.0 ± 5.67	6.56 ± 2.4*

The values are the averages ± SD from three biological replicates. The significance of differences between the WT and Δ*sll0290* was evaluated by means of Student’s *t-test*, as to the polyP content or total P one under +S/++P conditions, or to the same factor under -S/++P conditions. *P<0.05.

We then overexpressed *sll0290* in *E. coli* cells to investigate its function more directly (**Figure 3D**). SDS-PAGE analysis of total cellular proteins showed that IPTG induced expression of a protein with a molecular weight of 80 kDa in *E. coli* cells transformed with the *sll0290*-overexpression vector, but not in those with the empty vector (left panel, **Figure 3D**). The size of the induced protein coincided well with the postulated molecular weight of the product of introduced *sll0290*. Fluorescence-microscopic images exhibited abundant DAPI-stained polyP bodies in the *sll0290*-overexpressing cells, however, polyP body absent in the control, which indicated that *sll0290* can force *E. coli* cells to hyperaccumulate polyP. Collectively, it was proved that *sll0290* represents *ppk1* by demonstrating the loss of polyP accumulation ability in *Synechocystis* Δ*sll0290* and gain of this ability in the *E coli sll0290*-overexpressing transformant, together with the structural characterization of Sll0290 as Ppk1.

### Responsibility of *ppk1* for determination of the level of cellular P utilization

**Table 1** shows that total P contents were 6.79 and 3.12 fmol P·cell^-1^ in the WT and Δ*ppk1*, respectively, under +S/++P conditions, indicating a 2.2-fold lower level in Δ*ppk1*. However, Pi contents were almost equally low for the WT and Δ*ppk1* (0.45 and 0.75 fmol Pi·cell^-1^, respectively). These results suggested that WT cells require *ppk1* not only for polyP accumulation at a steady-state low level but also for the accumulation of large amounts of other P metabolites. Under -S conditions, WT cells increased the total P content by 5.3-fold, while maintaining Pi at a low level. Notedly, polyP bodies explained only a small portion of the -S-induced increase in the total P content in the WT cells (**Table 1**; 6.9 and 29.2 fmol P·cell^-1^ increases in polyP and total P, respectively). Relative to WT cells, Δ*ppk1* ones showed repression in the -S-inducible increase in the total P content (only 3.4 fmol P·cell^-1^ increase, c.f., 29.2 fmol P·cell^-1^ increase in the WT). These results underscored the significant role of *ppk1* in the -S-induced increase in P-metabolite contents, i.e., P-utilization level, including polyP hyperaccumulation as a small portion.

### Defects in cell survival in Δ*ppk1* under -S conditions

Corresponding to cell growth in Δ*ppk1* under +S conditions, comparable to that in the WT (**Figure 2B**), exponentially growing cells showed no obvious defects in ongoing growth after their shift to a fresh BG11 agar plate (see 0 h in **Figure 2B** inset). However, at the mid-stationary phase (see 48 h in **Figure 2B** inset, OD_730_ = 2.0), Δ*ppk1* cells, distinct from WT ones, became seriously impaired in their subsequent growth or survival on the agar plate. These results, together with the inability of Δ*ppk1* cells to hyperaccumulate polyP bodies upon entry into the stationary phase (**Figure 2D**), demonstrated the crucial role of *ppk1*-dependent polyP body accumulation for cellular acclimation to ambient stresses imposed during the stationary phase, such as the loss of nutrients like S. Physiological processes for acclimation to -S in place of -S/++P were then investigated in Δ*ppk1* to gain deep insights into the decreased survival ability of its stationary cells. Fluorescence-microscopic observations showed that polyP bodies increased in WT cells with -S at 5 h, followed by continuing increase until at 24 h (**Supplementary Figure 1**). In contrast, polyP bodies remained undetectable in Δ*sll0290* cells even under -S conditions (**Supplementary Figure 1**). It was therefore demonstrated that -S as well as -S/++P induces *ppk1*-dependent polyP body hyperaccumulation in *Synechocystis*, and that S-limitation would be one of the triggers for induction of polyP hyperaccumulation in the early stationary cells (**Figure 2D**).

**Figure 4:**
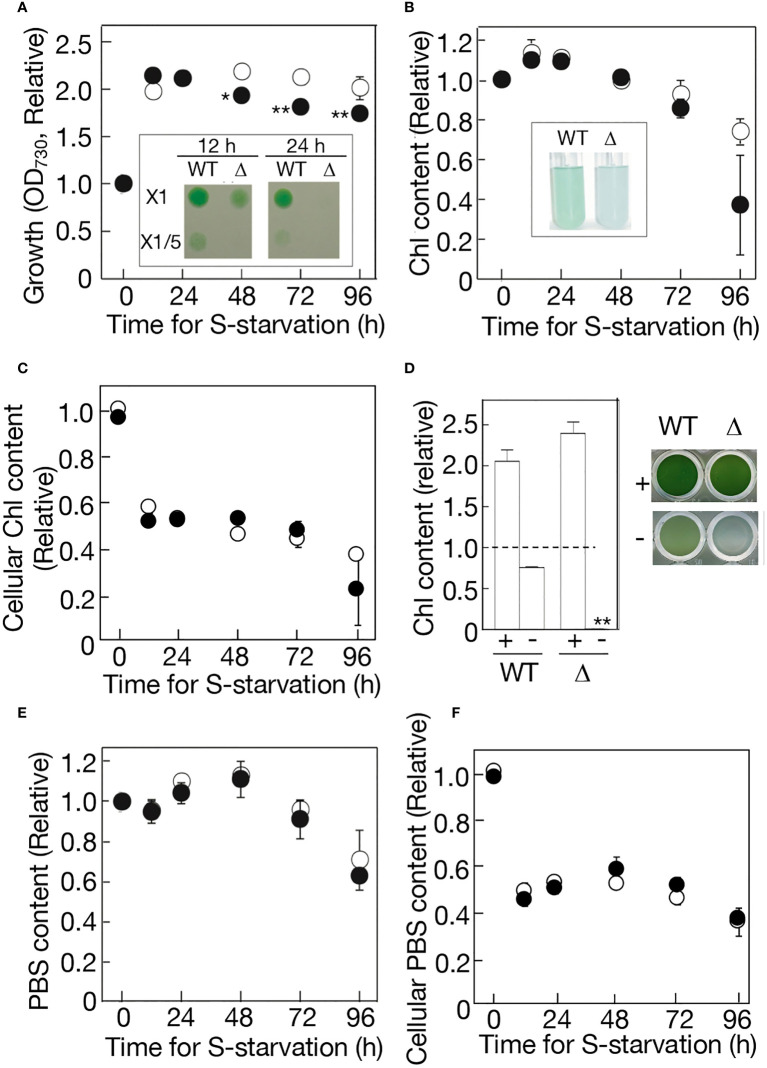
Effects of *ppk1* disruption on physiological responses to -S. **(A)** Cell growth with aeration of ordinary air, which was measured by OD_730_ values in the culture. WT and Δ*ppk1* values are shown relative to that at 0 h in the WT (0.293). Inset, cell viability on the BG11 agar plate. The liquid cultures at 12 h and 24 h were diluted to OD_730_ = 0.1 in the BG11 medium, thereafter, and 10 μL of the diluted cultures were placed on an agar plate for subsequent cell growth. **(B)** Chl contents in the batch culture. WT and Δ*ppk1* values are shown relative to that at 0 h in the WT (2.13 μg Chl·mL^-1^). Inset, the photograph of -S cultures at 96 (h) **(C)** Chl contents per OD_730_·mL culture, which were estimated through division of values in **(B)** by those in **(A)**. WT and Δ*ppk1* values are shown relative to that at 0 h in the WT (7.27 μg Chl· OD_730_^-1^·mL^-1^). **(D)** Quantitative behavior of Chl in the standing culture in a titer plate under ordinary air. Left, Chl contents in 7-day standing culture of the WT and Δ*ppk1*. + and - indicate culturing with and without external S-source, respectively. WT and Δ*ppk1* values are shown relative to those at 0 h in the WT and Δ*ppk1*, respectively (2.09 and 1.88 μg Chl·mL^-1^ in WT and Δ*ppk1*, respectively). Right, the photograph of the standing-culture in the titer plate. **(E)** PBS contents in the batch culture. WT and Δ*ppk1* values are shown relative to that at 0 h in the WT (0.0821). **(F)** PBS contents per OD_730_·mL culture, which was estimated through division of values in **(E)** by those in **(A)**. WT and Δ*ppk1* values are shown relative to that at 0 h in the WT (0.280 OD_730_^-1^·mL^-1^). Open circles represent WT, while closed circles represent Δ*ppk1*. The values indicated in **(A–F)** are the averages ± SD from three biological replicates, with some SD bars hidden within symbols. The significance of differences in **(A)** regarding the Δ*ppk1* values at 48-96 h relative to that at 0 h, and that in **(D)** regarding the Δ*ppk1* value relative to WT one under -S conditions, was evaluated by means of two-sided Student’s *t*-test. **P<0.01. *P<0.05.

Cell growth under -S stress in aeration-liquid culture was then compared between the WT and Δ*ppk1* (**Figure 4A**). Consistent with our previous report (Hirai et al., 2019), WT cells showed only a two-fold increase in the first 12 h of -S, and thereafter, the increased level remained almost unaltered until at 96 h. Δ*ppk1*, like the WT, achieved a two-fold elevation in cell growth at 12 h, which, however, was followed by a slight but definite decrease, distinct from the WT (**Figure 4A**). Importantly, Δ*ppk1* cells grown under -S conditions for 12 h, compared to the WT counterparts, were impaired in survival after their shift to an agar plate containing normal +S medium (see **Figure 4A** inset). The impairment in cell survival became more serious in Δ*ppk1* with the -S-culturing period extended to 24 h. The defect of Δ*ppk1* in cell survival at the early-stationary phase might be triggered at least partially by a loss of external S-source in the culture.

Regarding photosynthetic pigments, the WT kept Chl at an almost unaltered level in the first 72 h, and then degraded it mildly to 74% of the initial level at 96 h (**Figure 4B**). Δ*ppk1* also kept Chl at almost the same level in the first 72 h, thereafter, showing a trend to degrade it more drastically than the WT to as low as 37% at 96 h. As a result, the cellular content of Chl decreased similarly for WT and Δ*ppk1* in the first 12 h (**Figure 4C**), mainly through cell-growth dependent Chl dilution (**Figure 4A**), thereafter kept at the decreased level until at 72 h. However, in the next 24 h, the accelerated Chl degradation in Δ*ppk1*, relative to in the WT, resulted in a steeper decrease in the cellular Chl content on average in Δ*ppk1* than in WT (**Figure 4C**). The Chl degradation in Δ*ppk1* at later times was obvious when the cells were standing-liquid cultured in a microtiter plate: -S culturing for 7 days caused Chl in the culture to be degraded to 76% of the initial level in the WT, and to almost zero in Δ*ppk1*, although +S culturing enabled the Chl content to increase by ca. 2-fold in both the WT and Δ*ppk1* (**Figure 4D**).

Meanwhile, PBS demonstrated almost indistinguishable quantitative behavior for the WT and Δ*ppk1* in the aeration-liquid culture: PBS was maintained almost at the initial level in the first 72 h, followed by a decrease to 63-71% of the initial levels in the next 24 h (**Figure 4E**). Accordingly, WT and Δ*ppk1* showed a similar decreasing pattern in the cellular content of PBS (**Figure 4F**). It therefore seemed that the functioning of *ppk1* for polyP hyperaccumulation is necessary for -S cells at the early phase to properly maintain physiological fitness, and also for -S cells at the later phase to avoid intensive degradation of the photosystem complexes I and/or II.

## Discussion

### Identification of *ppk1* in *Synechocystis*

A substantial amount of information has accumulated regarding the cyanobacterial homologs of *ppk* and *ppx* over the past nearly three decades through the determination of the genomic DNA sequences in more than 200 cyanobacterial strains (CyanoCyc, https://cyanocyc.org/). However, little progress has been made in the functional identification of genes involved in polyP metabolism or their physiological significance. This lack of progress can be attributed largely to only a few successful reports in genetic manipulation of polyP metabolism in cyanobacteria, such as Δ*ppk1* in *Synechococcus* (Gómez-García et al., 2013) and Δ*ppx* in *Synechocystis* (Hiyoshi et al., 2021).

Regarding polyP synthesis, here, Sll0290 in *Synechocystis* exhibited primary structural features characteristic of known Ppk1 proteins (**Figure 1**). Consistently, *sll0290* was functionally identified as *ppk1* through loss-of-function analysis in *Synechocystis* using chemical-quantitative, fluorescence and electron microscopy, and *in vivo* NMR techniques (**Table 1**; **Figures 3A–C**), and also through gain-of-function analysis in *E. coli* (**Figure 3D**). It was demonstrated that *Synechocystis ppk1* is responsible for the main part of polyP body accumulation at a low level under +S conditions (**Table 1**; **Figure 2D**), and also for -S or stationary-phase induced hyperaccumulation of polyP bodies and/or soluble polyP (**Table 1**; **Figures 2D**, **3**; **Supplementary Figure 1**). The low levels of polyP detected in Δ*ppk1* (**Table 1**) might be synthesized by the action of other polyP metabolic genes, including *ppk2* slr1363 (Wang et al., 2018).

Gómez-García et al. (2013) identified *ppk1* in *Synechococcus* through its protein structural and functional characterization. Particularly, the functional identification of *ppk1* relied on observations of disruptant defects in polyP accumulation at a low steady-state level and an elevated level during the logarithmic and stationary phases, respectively, using techniques of enzymatic polyP quantitation and/or electron microscopy. During the preparation of our manuscript, Sebesta et al. (2024) recently reported the functioning of *sll0290* as *ppk1* through the observations of Δ*ppk1* defects in polyP overplus using polyP quantitation. Therefore, we have identified *ppk1* in *Synechocystis* not only through stationary-phase-inducible but also through a distinctive -S-inducible polyP hyperaccumulation phenomenon and by obtaining more cumulative evidence using diverse techniques.

### Non-essentiality of *ppk1* for cell growth in *Synechocystis* under normal conditions

This study demonstrated that *ppk1* is dispensable for cell growth in *Synechocystis* under ordinary-air aeration (**Figure 2B**). Similar results were presented by Sebesta et al. (2024); we further concluded that the dispensability of *ppk1* stems from its lack of involvement in the construction or functionality of the photosynthetic machinery (**Figure 2C**). These results, combined with the lack of requirement for *ppx* in *Synechocystis* (Hiyoshi et al., 2021), suggested that both *ppk* and *ppx*, i.e., main players in polyP metabolism, are not necessary for cell growth in *Synechocystis* under low-CO_2_ conditions. However, it should be emphasized that this lack of requirement holds true in logarithmic cells but not in stationary cells (**Figure 2B** inset, see below). Meanwhile, Gomez-Garcia et al. (2003) previously proposed the responsibility of *ppk1* for carboxysome biogenesis in *Synechococcus*, based on the high-CO_2_ requiring phenotype and increased levels of mRNAs of the genes for carboxysome construction in *Synechococcus* Δ*ppk1*. The *Synechococcus* strain was isolated from microbial mats in extreme and nutrient-limited environments of an alkaline hot spring (Allewalt et al., 2006). The physiological roles of *ppk1* might have developed in species-dependent manners in cyanobacteria through their evolutional diversification. PolyP bodies were often positioned in the vicinity of carboxysomes in some cyanobacteria (Iancu et al., 2010; Moura et al., 2019). Future studies will aim to elucidate what determines whether or not *ppk1* is responsible for carboxysome biogenesis in cyanobacteria: specific properties of polyP bodies and/or Ppk1, or other factors associated with them?

### Essentiality of *ppk1* for cellular acclimation to ambient stresses in *Synechocystis*

In non-photosynthetic microorganisms, it has been demonstrated, through genetic manipulation of *ppk1*, that *ppk1*-dependent polyP synthesis is crucial for cellular acclimation to ambient stresses, such as entry into the stationary phase, nutrient depletion, heat, oxidants, and hyperosmosis (Rao and Kornberg, 1996; Kim et al., 1998; Kuroda et al., 1999; Rao et al., 2009). Among these polyP-synthesis-dependent stress-acclimation responses, those that accompanying polyP hyperaccumulation would convincingly represent the importance of polyP synthesis. In cyanobacteria, polyP hyperaccumulation can be observed under stationary-phase, -S, or -N conditions, or as an overplus phenomenon (Harold, 1966; Grillo and Gibson, 1979; Lawry and Jensen, 1979; Lawrence et al., 1998; Gómez-García et al., 2013 Sebesta et al., 2024). However, information is limited on the gene(s) responsible for polyP hyperaccumulation and its physiological significance in cyanobacteria: polyP hyperaccumulation, as mentioned above, was demonstrated to depend on *ppk1* in only *Synechococcus* stationary cells (Gómez-García et al., 2013) and *Synechocystis* overplus ones (Sebesta et al., 2024), whereas the physiological significance of polyP hyperaccumulation, including these two cases, remains unclear. This study strongly suggested that *ppk1* is the gene responsible for polyP hyperaccumulation not only temporarily in early-stationary cells but also in -S cells in *Synechocystis* (**Table 1**, **Figures 2D** and **3A–C**, **Supplementary Figure 1**), and that *ppk1* is essential for physiological fitness in both stationary and -S cells (**Figures 2B** inset and **4A** inset, **4B, D**). It thus seems probable that *ppk1*-dependent polyP hyperaccumulation in early-stationary cells are triggered, at least partially, by -S stress. Considering the polyP hyperaccumulation in -S/++P cells as well as in -S cells, it would be improbable that polyP hyperaccumulation in -S cells was facilitated by a decrease in external-P source dependent on cell growth. This strengthens the hypothesis that -S triggers polyP hyperaccumulation in early stationary phase cells.

It was previously reported that prokaryotes lacking both *ppk1* and *ppk2* include vector-borne or obligate intracellular pathogens that rarely encounter ambient stresses, distinct from free-living prokaryotes (Zhang et al., 2002; Wang et al., 2018). Wang et al. (2018) further observed that the presence of polyP-metabolism genes, including *ppk1*, correlated positively with bacterial proteome size and the number of virulence genes, suggesting a potential relationship of polyP in bacterial life style and environmental durability. Consistently, among cyanobacteria lacking both *ppk1* and *ppk2* are a sponge symbiont *Candidatus Synechococcus spongiarum* and diatom symbionts *Richelia intracellularis* HH 01 and *Richelia intracellularis* HM 01 (**Supplementary Table 1**; Usher, 2008; Hilton et al., 2013). Together, these observations suggested that *ppk1*-containing cyanobacteria, as well as non-photosynthetic microorganisms containing *ppk1*, may utilize polyP for cellular acclimation to ambient stresses such as nutrient limitation. In this context, it is plausible that in *Synechococcus* as well as in *Synechocystis*, *ppk1*-dependent polyP hyperaccumulation in stationary phase cells could be induced by nutrient loss stresses, including -S. Comparative studies using *Synechocystis* and *Synechococcus* Δ*ppk1* mutants to assess responses to other stresses such as heat, oxidants, and hyperosmosis would help elucidate the roles of *ppk1* further.

Future studies will investigate how physiological fitness is properly maintained through polyP hyperaccumulation in -S cells. Additionally, they will explore what stresses, including the possibility of -S, trigger *ppk1*-dependent polyP hyperaccumulation in early-stationary cells, and how physiological fitness is properly maintained in stationary cells where the hyperaccumulated polyP is degraded. In this context, it is important to understand what physiological disadvantages manifest in Δ*ppk1*, relative to the WT, under -S conditions. As a physiological response to -S, WT and Δ*ppk1* showed a similar quantitative decrease in Chl or PBS in the first 72 h (**Figures 4C, F**). The decrease would indicate that the size of the photosynthetic machinery was properly downregulated in Δ*ppk1* as well as in WT, to repress the generation of photosynthesis-derived reactive oxygen species (ROS) (Schwarz and Forchhammer, 2005; Latifi et al., 2009). Physiological unfitness began to appear in Δ*ppk1* as early as at 12 h under -S conditions (**Figure 4A** inset), reflecting injuries in some other physiological process than the photosynthetic size reduction. In a green alga, *Chlamydomonas reinhardtii*, a polyP-deficient mutant was found to have a disruption in the gene for polyP-synthesis catalytic subunit of the vacuolar transporter chaperon (VTC) (Sanz-Luque et al., 2020b). The mutant characterization then revealed that -S-induced polyP accumulation is vital for controlling ATP homeostasis to repress disordered photosynthetic and respiratory electron flows, and accordingly is essential for cellular acclimation to -S conditions (Sanz-Luque et al., 2020b). Our study revealed that in *Synechocystis* cells, *ppk1* is necessary not only for determination of the polyP content but also for promoting the accumulation of total P metabolites or enhancing P-utilization level under +S conditions, with this trend particularly strengthened under -S conditions (**Table 1**). P-metabolites that require *ppk1* for high P-utilization levels would include RNA, in particular, and DNA, which represented 62 and 15% of the total P in *Synechocystis* +S cells (Hiyoshi et al., 2024). Meanwhile, -S-inducible P-metabolic regulation, including that of cellular energization, might become so malfunctional in Δ*ppk1* as to cause early-phase defects in physiological fitness. However, this hypothesis awaits experimental validation.

In summary, this study demonstrated that *ppk1* contributes to the generation of the majority of polyP in *Synechocystis* cells, regardless of whether they are polyP-hyperaccumulating under -S or early-stationary conditions or have low polyP accumulation under +S conditions. Additionally, it showed that *ppk1* is essential for physiological fitness in -S or stationary cells. It was therefore likely that polyP hyperaccumulation in early-stationary cells are at least partially triggered by concurrent S limitation. Besides, it appeared that *ppk1* promotes P utilization in *Synechocystis* cells to maintain its proper levels not only through polyP synthesis but also through extensive involvement in the regulation of other P metabolism pathways. Our study contributes to the construction of a fundamental framework for a comprehensive understanding of the *ppk1*-dependent mechanism by which cyanobacterial cells acclimate to nutrient-depletion stresses, including those encountered during the stationary phase of culture.

## Data availability statement

The raw data supporting the conclusions of this article will be made available by the authors, without undue reservation.

## Author contributions

NS: Writing – review & editing, Writing – original draft, Validation, Supervision, Methodology, Investigation, Funding acquisition, Conceptualization. ME: Writing – review & editing, Validation, Methodology, Investigation. HN: Writing – review & editing, Validation, Methodology, Investigation. SF: Writing – review & editing, Validation, Methodology, Investigation, Conceptualization. MT: Writing – review & editing, Validation, Methodology, Investigation, Conceptualization.
