# Supplementary material for: Polyphosphate-kinase-1 dependent polyphosphate hyperaccumulation for acclimation to nutrient loss in the cyanobacterium, Synechocystis sp. PCC 6803
Source: Front Plant Sci. 2024 Jul 31;15:1441626. doi: 10.3389/fpls.2024.1441626 (PMC11322815; doi:10.3389/fpls.2024.1441626)
Supplement: Supplementary file 1 [file Image_1.pdf]

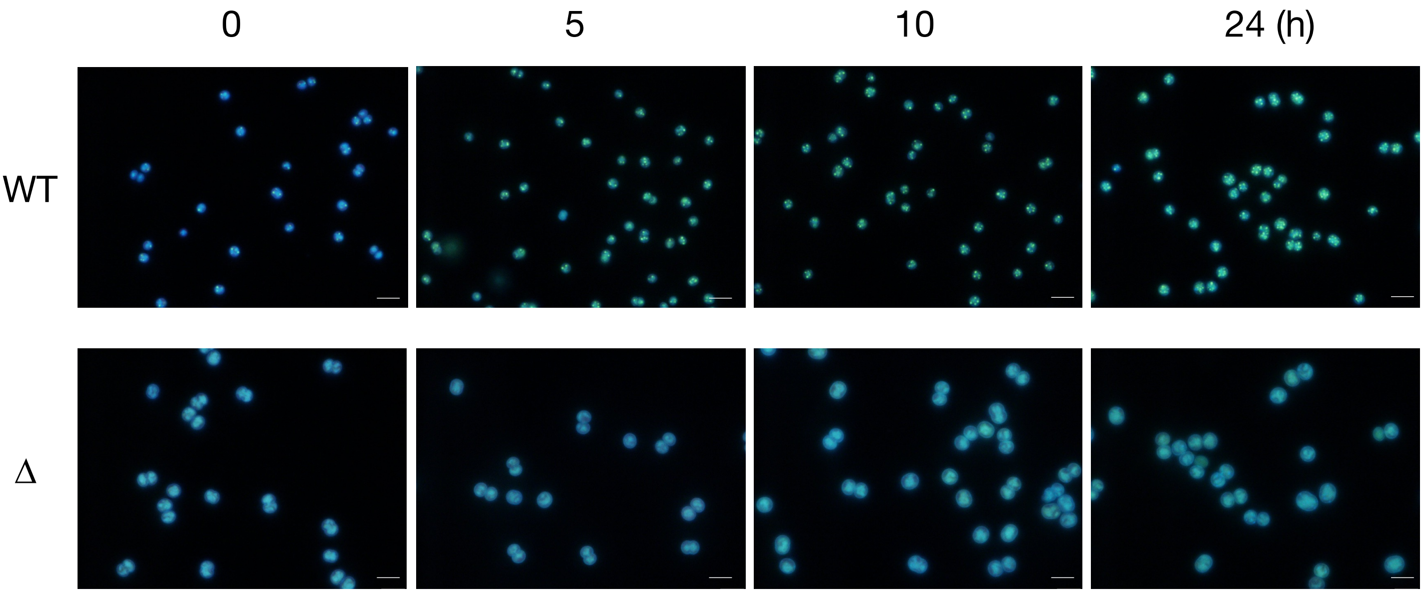


Supplemental Fig. 1. Fluorescence-microscopic images of the WT and Δ*ppk1* cells. Cells grown under –S conditions were DAPI-stained at indicated times for observation of polyP bodies.
